# *N*-Acetylcysteine: more than preventing contrast-induced nephropathy in uremic patients—focus on the antioxidant and anti-inflammatory properties

**DOI:** 10.1007/s11255-022-03455-3

**Published:** 2023-01-03

**Authors:** Dainora Cepaityte, Konstantinos Leivaditis, Garyfallia Varouktsi, Athanasios Roumeliotis, Stefanos Roumeliotis, Vassilios Liakopoulos

**Affiliations:** 1 Dialysis Unit, General Hospital of Serres, 62120 Serres, Greece; 2 Division of Nephrology and Hypertension, 1st Department of Internal Medicine, School of Medicine, AHEPA Hospital, Aristotle University of Thessaloniki, 54636 Thessaloniki, Greece

**Keywords:** Oxidative stress, Antioxidants, Chronic kidney disease, End-stage renal disease, Inflammation, *N*-Acetylcysteine, Renal replacement therapy

## Abstract

Oxidative stress (OS) has been recognized as a pathophysiologic mechanism underlying the development and progression of chronic kidney disease (CKD). OS, which results from the disturbance of balance among pro-oxidants and antioxidants favoring the pro-oxidants, is present even in early CKD and increases progressively along with deterioration of kidney function to end-stage kidney disease (ESKD). In ESKD, OS is further exacerbated mainly due to dialysis procedures per se and predisposes to increased cardiovascular morbidity and mortality. Therefore, since OS plays a pivotal role in the pathogenesis and progression of atherosclerosis in uremic patients, several strategies aiming to ameliorate OS in these patients have been proposed. Among those, *N*-acetylcysteine (NAC), a thiol-containing antioxidant agent, has attracted special attention due to its pleiotropic functions and beneficial effect in various OS-related entities including paracetamol overdose and prevention of contrast-induced nephropathy. In this review, we present the currently available literature on the antioxidant and anti-inflammatory properties of NAC in CKD, including hemodialysis and peritoneal dialysis.

## Introduction

Oxidative stress (OS) results from the disruption of balance between pro-oxidants (substances gaining electrons) and antioxidants (substances donating electrons) weighing in favor of the former. This balance is essential for maintaining homeostasis, and when disrupted, may lead to multiple pathological conditions, including cancer and atherosclerosis. Free radicals, including hydroxyl, superoxide anion, hydrogen peroxide, oxygen singlet, nitric oxide and peroxynitrite are independent molecular species that contain unpaired electrons in an atomic orbital. Due to their molecular structure missing electrons, free radicals are unstable and highly reactive. In an attempt to gain stability, free radicals interact and “steal” one electron from macromolecules such as nucleic acids, proteins, lipids and carbohydrates, resulting in their structural oxidative modification and dysfunction [1–4]. Antioxidants, on the other hand, are stable molecules that donate electrons and neutralize free radicals minimizing cellular damage. Naturally occurring antioxidant defense mechanisms might be either enzymatic (dismutase superoxide, catalase, and glutathione peroxidase) or non-enzymatic (uric acid, ascorbic acid, bilirubin, albumin, flavonoids, α-tocopherol, ubiquinol and carotenoids) [2, 4, 5]. Although we tend to refer to OS as a harmful condition, when maintained at low levels, free radicals are essential for human health and thus, low-level OS is crucial for maintaining homeostasis and plays a pivotal role in redox signaling, cell metabolism, immune defense, neural activity and cell reproduction.

The leading cause of mortality in chronic kidney disease (CKD) patients remains cardiovascular (CV) disease [6], which is partially attributed to OS. Compared to healthy individuals, OS along with inflammation are highly prevalent even at early stages of CKD and are gradually increased parallelly to deterioration of kidney function, as disease progresses towards end-stage renal disease (ESRD) [2, 7]. In the uremic environment, elevated OS leads to reduced bioavailability of nitric oxide (NO) resulting in decreased vascular relaxation, vascular damage, lipid peroxidation and subsequently endothelial dysfunction, the hallmark of atherosclerosis [7, 8]. The increase of OS in CKD is also attributed to the limited activity or reduced levels of antioxidants, most commonly resulting from nutritional restrictions regarding fruits and vegetables [9–11]. Compared to non-dialysis ESRD, those undergoing maintenance hemodialysis (HD) present significantly increased OS status. This is due to several factors. The HD procedure per se aggravates OS status; during a dialysis session, reactive oxygen species (ROS) accumulation begins immediately, peaking at 3 h to a 14-fold increase and decreases to pre-dialysis levels shortly after the end of the session [12]. The generated free radicals interact with multiple biomolecules altering their structural and functional integrity [11]. In addition, the protein-binding properties of multiple uremic toxins limit their removal via HD, promoting endothelial damage, further inflammation and OS generation [6]. Other factors promoting free radicals formation during a HD session are arteriovenous fistulae dysfunction, use of central venous catheters, contamination of the dialysate and intravenous administration of iron and heparin [13]. Anti-oxidant defense systems are also reduced in HD patients and contribute to the increased levels of OS [14].

Although peritoneal dialysis (PD) is considered a more compatible dialysis technique compared to HD, OS is still present in this dialysis modality and is associated with clinical adverse endpoints [13]. In PD, the mechanisms triggering OS differ significantly from HD [3, 15, 16] and are mainly attributed to the composition of low pH, lactate-buffered, hyper-osmolar and hyperglycemic PD solutions. The process of PD fluids’ heat sterilization leads to the formation of glucose degeneration products (GDP) which promote generation of advanced glycation end-products (AGEs) and pro-oxidants [17–20]. Chronic exposure of the peritoneal membrane to AGEs and ROS leads to progressive increase of peritoneal vascular permeability and cellular apoptosis [19]. These molecular and structural alterations eventually result to the occurrence of adverse clinical endpoints, including loss of residual renal function, inflammation, peritonitis, technique failure, endothelial dysfunction, atherosclerosis, CV disease and mortality [16, 21–23]. Since the main culprit for OS in PD is PD solutions, the strategies to reduce OS in these patients include the use of more biocompatible fluids with neutral pH, low glucose generation products with bicarbonate as buffer. In addition, volume management and strict glycemic control might also help using solutions with lower glucose concentrations [19, 24–28].

In ESRD patients undergoing either HD or PD, OS is increased and associated with adverse events, including development and progression of atherosclerosis, CV disease and mortality [29–48]. Therefore, there is a need for new strategies to ameliorate OS in these patients and possible protect them from CV disease. During the past decade, *N*-acetylcysteine (NAC) has emerged as a novel and quite powerful antioxidant agent [49]. Here, we aim to review the existing data regarding the possible antioxidant and anti-inflammatory properties of NAC in CKD and ESRD.

### NAC: molecular structure and properties

**Fig. 1:**
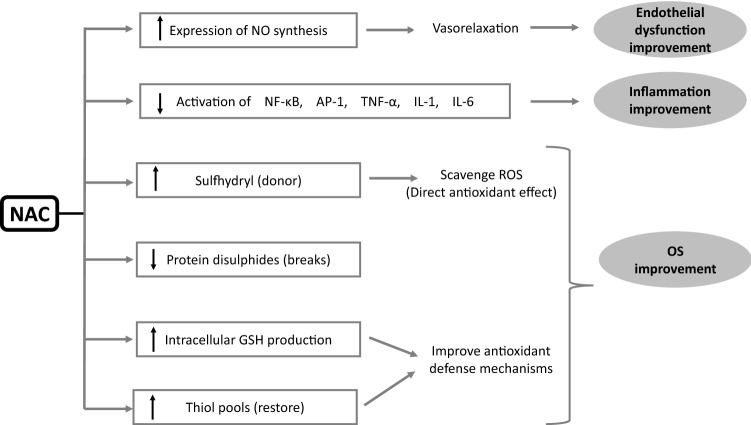
Anti-inflammatory and antioxidant molecular mechanisms of NAC

NAC was first used in the early 1960s as a mucolytic agent in patients with cystic fibrosis. The acetylation of the *N*-terminal of cysteine provides adequate stability to the sulfur-containing molecule of cysteine to deliver a thiol group (reduced sulfhydryl moiety) and allows it to function as a mucolytic agent by disrupting the disulfide bridges within the glycoprotein matrix of mucus without being deactivated by metabolism and rapid oxidation in the solution [50]. NAC has been also used as an effective antidote in paracetamol overdose acting as a precursor of the substrate (l-cysteine) in synthesis of hepatic glutathione (GSH) which might be depleted due to conjugation with paracetamol. GSH is the most important intracellular, endogenous antioxidant comprising of glutamic acid (E), glycine (G), and cysteine (C). The rate of GSH synthesis depends on the activity of glutamate-cysteine ligase. GSH has multiple functions including protein thiolation, drug detoxification and antioxidative protection of cellular components. The antioxidative properties of GSH derive from the free sulfhydryl group that directly interacts with free radicals as well as its role as a substrate of co-factor for various enzymes including glutathione reductase, glutaredoxin, glyoxalases 1 and 2, glutathione transferase, and membrane-associated proteins with divergent functions in Eicosanoid and Glutathione metabolism (MAPEG) [51, 52]. The anti-inflammatory and antioxidant molecular mechanisms of NAC are shown in Fig. 1.

During the past decade, research has focused on the possible beneficial antioxidant effects of NAC in multiple conditions where OS is involved [50]. NAC is believed to act as an antioxidant by several mechanisms: first, it is a direct sulfhydryl donor for the neutralization of ROS; second, it modulates extracellular glutamate and intracellular GSH levels, third, it acts as a reducing agent for protein disulfides and finally it restores thiol pools, which in turn regulate the redox state [7, 53–56]. In addition to antioxidant properties, NAC inhibits the function of pro-inflammatory transcription factors such as AP-1 (activator protein 1) and NF-κB (nuclear factor kappa-light-chain-enhancer of activated B cells), well-known pre-cursors of OS [57, 58]. In addition, NAC is believed to exert cardioprotective properties through increasing endothelial nitric oxide synthase expression, improving nitric oxide bioavailability and suppressing angiotensin-converting enzyme activity, thus leading to vasorelaxation [7, 59–64]. Furthermore, NAC acts as a methyl donor in the conversion of homocysteine to methionine and also contributes to the displacement of homocysteine from serum albumin binding sites, a property that can be utilized during dialysis sessions to increase levels of unbound homocysteine available for plasma clearance [65–70].

### NAC for the prevention of contrasted-induced nephropathy

Besides its use as a mucolytic agent, NAC has been widely used for the prevention of contrast-induced nephropathy (CIN) as various evidence suggest the involvement of OS in the pathophysiology of this condition [2, 71, 72]. The use of NAC as a preventive measure for the development of CIN relies on its antioxidant and vasorelaxant properties; NAC reduces ROS and tissue damage in the kidneys, minimizes vasoconstriction and stabilizes renal hemodynamics [71, 73]. To investigate the beneficial effect of NAC on CIN prevention, Guo et al. [74] conducted a meta-analysis including seven randomized clinical trials and 1710 ST segment elevation myocardial infarction patients undergoing primary percutaneous coronary intervention and demonstrated a 49% and 63% reduced risk of CIN and all-cause in-hospital mortality, respectively. In a subgroup analysis, the preventive effect of NAC appeared greater in patients with pre-existing impaired renal function and in those receiving higher dosages of NAC. Similarly, other meta-analyses coherently reported a 22–33% beneficial effect of NAC on preventing CIN [73, 75, 76], which was more pronounced in patients with pre-existing CKD [71]. However, the largest RCT until to date, the PRESERVE trail, failed to show any therapeutic effect of NAC regarding CIN prevention [77] and other meta-analyses providing conflicting results 12/27/2022 4:41:00 P.M. Based on the contradictory results of the existing trials and meta-analyses, current guidelines do not longer recommend NAC for CIN prevention. Since the alternatives for CIN prevention are very limited, future trials are needed examining different dosages and timing of NAC administration, combined with saline hydration in order to draw definite conclusions regarding the reno-protective effects of NAC.

### NAC as an antioxidant in CKD

Accumulating preclinical data support the use of NAC in uremia and CKD. In animal models, NAC prevented GSH depletion in vascular cells exposed to uremic serum and thus diminished systemic OS that promotes CKD progression [78]. In addition, in a model of uremia-enhanced atherosclerosis, NAC reduced the progression of atheroma also by reducing OS [79]. In other in vivo studies, NAC appeared to have a protective effect on cyclosporin induced nephrotoxicity, through amelioration of local and systemic OS [80]. Experimental studies also suggested another molecular pathway through which NAC combats OS; in uremic animals, NAC administration directly attacked and neutralized AGEs that are released due to the uremic environment [81].

The clinical data regarding the effect of NAC in CKD populations are limited and have failed to show any reno-protective effect. Short-term oral NAC administration in CKD patients stage 3 showed no difference in renal function compared to placebo [82–84]. Similarly, NAC administration failed to show any therapeutic effect on the proteinuria levels of CKD patients with [85] and without diabetes [53]. However, in a cohort of CKD patients (stages 3–4) that received intravenous iron infusion for anemia correction, NAC resulted in a significant reduction of OS [86]. In kidney transplant recipients, the data are extremely limited; only a double-blinded, placebo-controlled randomized controlled trial (RCT) has been performed until to date [9]. This study showed a significant reno-protective effect in the NAC group, assessed by improvement in immediate graft function (28% increase over placebo) and first week eGFR (14 ml/min higher than placebo). Interestingly, this reno-protective effect of NAC was not attributed to its’ antioxidant properties, since there was no difference on malondialdehyde (MDA) levels between the groups. The authors hypothesized that other NAC properties, such as anti-inflammatory and vasodilatory might be responsible for their findings. To investigate the possible clinical benefits of NAC supplementation, Ye et al., performed a recent meta-analysis [87] including 768 CKD patients and 20 studies and found that NAC was safe without any severe adverse events. Moreover, NAC suppressed the levels of inflammatory cytokines and homocysteine, protected kidney function and was associated with reduced CV events (relative risk = 0.60, number needed to treat = 5.29). However, the authors recognized as limitations of their study the heterogeneity and low quality of the included studies and the fact that the majority of the pooled data included only few trials.

Therefore, the majority of data supporting the antioxidant effects of NAC in CKD are derived from experimental studies. The clinical studies are very scarce and have failed to show a clear-cut clinical benefit of NAC supplementation in pre-dialysis CKD.

### NAC as an antioxidant in HD

Advanced oxidation protein products (AOPPs) in uremic plasma are indicators of oxidative damage to proteins and act as inflammation mediators resulting in monocyte and polymorphonuclear (PMN) activation. Release of AOPPs promotes monocyte respiratory burst and tumor necrosis factor-a (TNF-a) synthesis while PMNs produce free radicals by the molecular pathway of nicotinamide adenine dinucleotide phosphate (NADPH) oxidase and myeloperoxidase (MPO). NAC inhibits AOPP-induced oxidation of both monocytes and PMNs in a receptor-dependent way; therefore, it is suggested that in HD, NAC’s antioxidant activity might be selective and dependent on intracellular signaling rather than nonspecific oxidant scavenging [88–90]. A clinical trial in 24 chronic HD patients evaluated the circulating levels of MDA (a lipid peroxidation marker formed in the tissues by exposure to free radicals) pre- and post-dialysis after NAC administration (600 mg per os twice daily for 4 weeks) demonstrating that NAC significantly reduced the levels of MDA compared to placebo. Of note, HD alone was not able to diminish elevated MDA levels in chronic HD patients suggesting that glutathione repletion by NAC might be an additional mechanism contributing to the antioxidant properties of NAC [91]. Besides attacking directly and neutralizing free radicals, a double-blind, placebo-controlled RCT supported that NAC administration might reduce OS in chronic HD patients also by restoring the antioxidant defense mechanisms, assessed by total antioxidant capacity (TAC) [2].

In HD patients, intravenous iron administration is frequent and associated with increased OS. A randomized, cross-over clinical trial divided 40 HD patients in four cross-over treatment groups of 10 patients each according to iron sucrose administration dose (50 or 100 mg) and NAC supplementation (NAC or no NAC). NAC administration resulted in significant increase in TAC, whereas MDA serum levels were only reduced in the low iron dose group [92]. Swarnalatha et al. conducted a prospective, double-blinded, randomized controlled, cross-over study with 14 HD patients treated with intravenous iron receiving either NAC or placebo. NAC reduced MDA levels that were released after administration of intravenous iron therapy [5]. Likewise, another single-arm clinical trial reported decrease in MDA and asymmetric dimethylarginine (ADMA) levels post-intervention (NAC administration 600 mg per os before meals for 6 months) [93]. Since ADMA has been repeatedly associated with mortality and CV events in HD patients, another double-blind placebo-controlled clinical trial used it as a therapeutic target and showed that intravenous administration of high dose NAC (5 g) during HD resulted in significant reduction of serum ADMA levels post-dialysis compared with HD alone [94]. Since HD is a state of increased OS and inflammation, several studies aimed to investigate whether NAC supplementation might also ameliorate inflammation in these patients. A prospective, non-randomized, non-controlled clinical trial in a cohort of HD patients suggested a decrease in inflammatory and OS biomarkers after NAC administration, including high-sensitivity C-reactive protein (hs-CRP) and interleukin 6 (IL-6) [95], which have been repeatedly reported to be indicators of CV disease in CKD. IL-6 might act as a marker of atherosclerosis as well as a pro-atherogenic cytokine affecting multiple metabolic, endothelial, and coagulant pathways. In addition, CRP activates multiple inflammatory processes underlying the development of atherosclerosis [96–99]. Since CV morbidity and mortality in CKD has been associated with OS and inflammation [100], it was hypothesized that NAC might also exert cardioprotective effects in HD patients. A prospective, randomized, placebo-controlled trial in 134 maintenance HD patients, showed that daily, oral administration of NAC (600 mg/day) for a median of 14.5 months, was accompanied by a 40% reduction in the occurrence of CV events [101].

Another beneficial effect of NAC in HD patients is improvement of anemia. Red blood cell (RBC) reductase activity and TAC increased with NAC administration, while plasma levels of 8-isoprostane and oxidized low-density lipoprotein (ox-LDL) decreased, thus suggesting that positive outcomes of uremic anemia might be linked with improvement of OS status [55]. The presence of residual renal function (RRF) is an important predictor of survival in chronic dialysis patients [102–104]. In a non-randomized, non-blinded study, oral NAC significantly improved RRF in a small cohort of HD patients [105]. This was also confirmed in a randomized, multi-center, parallel-group, open-label study demonstrating that oral daily supplementation with NAC at a high dose of 1200 mg significantly improved RRF, urine volume and Kt/V [10].

Another novel risk factor for CV morbidity and mortality in CKD is hyperhomocysteinaemia [106]. Increased levels of homocysteine (Hcy) in plasma are indicators of increased OS, and contribute to endothelial dysfunction [8, 31]. Bostom et al., reported a non-significant reduction of homocysteine in a cohort of 11 HD patients receiving a single dose of oral NAC; however, the sample was very small and administration timing was not closely monitored to achieve optimal pharmacokinetics of NAC [107]. From this old study and there, several other investigators examined the effect of NAC on Hcy levels in ESRD patients. In a randomized, placebo-controlled cross-over study of 20 HD patients, Scholze et al., found that iv NAC administration during HD enhanced plasma Hcy clearance and ameliorated endothelial function [68]. Another study showed that addition of NAC to HD with high-flux membranes was accompanied with a significant reduction of circulating TNF-α, interleukin 10 (IL-10), hs-CRP and plasma Hcy, which was more pronounced in patients with RRF [108]. Thaha et al. performed a randomized, placebo-controlled trial and found a reduction in plasma Hcy and pulse pressure after dialysis with NAC supplementation [106]. Similarly, in a parallel, multi-center intervention study, Perna et al., showed that combined therapy of intravenous supplementation with NAC at a high dose of 5 g with 15 mg folates (5-methyltetrahydrofolate, MTHF) for 10 HD sessions, effectively reduced plasma Hcy levels in chronic HD patients [66]. The therapeutic effect of NAC in reducing plasma Hcy is reported to be about 11% higher than placebo [109].

In HD, NAC might improve OS, inflammation and anemia status; however, the existing evidence is derived from studies with various limitations, including short duration of treatment, small sample size and heterogeneity in the design. Moreover, the data regarding the clinical effect of NAC in hard endpoints, such as mortality and CV events are extremely limited, and therefore, currently, the administration of NAC in HD patients cannot be recommended. To elucidate whether NAC might be beneficial for CKD/ESKD patients and draw more definite conclusions, future, larger, well-designed RCTs are needed.

### NAC as an antioxidant in PD

Since the culprit for triggering OS in PD is PD solutions, it was interesting to hypothesize that addition of antioxidants, such as NAC, to the dialysate might improve OS status. Several experimental studies suggested the clinical stability of NAC in PD solutions [52, 110]. In vitro, generation of formaldehyde (which is toxic for the peritoneal membrane) in heat-sterilized PD solutions was reduced by the administration of reduced thiol compounds [111]. Administration of NAC in the high-glucose compartment of neutral-pH-type PD solutions prevented GDP-mediated peritoneal membrane failure in PD patients [52]. In addition, NAC appeared to reduce the generation of AGEs [112] and diminished mitochondrial oxidative injury induced by conventional peritoneal solution in human peritoneal mesothelial cells by preserving the levels of reduced glutathione [113, 114]. In uremic rat models undergoing PD treatment, NAC prevented the OS-induced structural and functional alterations of the peritoneal membrane [115], decreased inflammation and vascular injury and, therefore, preserved the integrity of the peritoneal membrane [116].

After the exciting results reported in experimental studies, several researchers designed clinical trials to explore if the beneficial effect of NAC in preclinical trials could be replicated in human subjects as well. A placebo-controlled study in PD patients found that oral intake of 600 mg of NAC twice daily for 8 weeks resulted in decreased plasma levels of IL-6 compared to controls [117]. Similarly, the administration of oral NAC significantly decreased hs-CRP levels in PD patients; this anti-inflammatory effect was more pronounced in patients with increased inflammatory status at baseline (CRP levels between 5 and 15 mg/L) [118]. Another placebo-controlled trial also examined the effect of NAC on inflammation status of chronic ambulatory PD subjects demonstrating that oral NAC administration (600 mg of NAC twice daily for 8 weeks) reduced the levels of several inflammatory biomarkers; interleukin 1 (IL-1), IL-6, hs-CRP, procalcitonin, complement C3, TNF-a and soluble intercellular adhesion molecule-1 (SICAM-1) [6]. Regarding clinical endpoints, Feldman et al. found in a small cohort of PD patients, that oral NAC (1200 mg twice daily for 4 weeks) significantly improved residual RRF [119]. Table 1 shows a summary of clinical trials investigating the use of NAC in CKD, HD and PD assessing its antioxidant and anti- inflammatory properties.

**Table 1:** Clinical trials of *N*-acetylcysteine (NAC) administration in chronic kidney disease (CKD), hemodialysis (HD) and peritoneal dialysis (PD) patients

Study ref	Year	Design	Population	Intervention	Outcome	Result
*CKD*						
Moist et al. [82]	2010	Double-blind, placebo-controlled RCT	60 CKD3 patients	4 doses of NAC (1200 mg) po at 12 h intervals	Plasma creatinine, eGFR, proteinuria, Cystatin C	No effect
Hasemi et al. [85]	2012	RCT	70 patients with diabetic nephropathy	600 mg × 2 NAC po + losartan 25 mg for 8 weeks	Proteinuria	No effect
Mainra et al. [83]	2007	Prospective	30 CKD3 patients	600 mg NAC po	Plasma creatinine, Cystatin C	No effect
Rehman et al.[84]	2008	Prospective	29 CKD3-5 patients	1200 mg × 2 NAC po for 2 days	Plasma creatinine, Cystatin C	No effect
Renke et al. [53]	2008	RCT, open-label, two-period cross-over	20 non-diabetic patients with proteinuria	1200 mg NAC po added to RAAS blockers for 8 weeks	Proteinuria	No effect
Agarwal et al. [86]	2004	Randomized, open-label, parallel	20 CKD3-4 patients receiving iron IV	600 mg × 2 NAC po for a week	Plasma MDA, ferritin, GSH, GSSG, SOD, GPX	Improvement in OS
*HD*						
Trimarchi et al. [91]	2003	Placebo-controlled RCT	24 HD patients	600 mg × 2 NAC po for 8 weeks	MDA levels	Improvement in OS
Thaha et al. [94]	2008	Double-blind RCT	40 HD patients	NAC 5 g IV during HD session	ADMA levels	Improvement in OS
Swarnalatha et al. [5]	2010	Double-blind, cross-over RCT	24 HD patients receiving iv iron infusion	600 mg × 2 NAC po for 10 days	MDA, TAC, hs-CRP,	Improvement in OS
Garcia-Fernandez et al. [92]	2010	Placebo-controlled, cross-over RCT	40 HD patients	2 g NAC IV 15 min before iron infusion	MDA, TAC	Improvement in OS
Tepel et al. [101]	2003	RCT	134 HD patients	600 mg × 2 NAC po	Major CV events	Improvement
Hsu et al. [55]	2010	Non-randomized, nested case–control	323 HD patients	200mgx3 NAC po for 3 months	Anemia	Improvement
Giannikouris [93]	2015	Prospective	48 HD patients	600mgx2 NAC po for 6 months	Hb, ADMA, MDA, MPO	Improvement of OS, inflammation and anemia
Saddadi et al. [95]	2014	Prospective	24 HD patients	600 mg × 2 po for 12 weeks	IL-6, hs-CRP	Improvement of inflammation
Feldman et al. [105]	2012	Prospective open-label, self-controlled	20 HD patients with RRF urine volume > 100 mL/d	1200mgx2 NAC po for 2 weeks	RRF, NO, ADMA	Improvement of RRF
Ahmadi et al. [10]	2017	Randomized, parallel-group, open-label	54 HD patients with RRF urine volume > 100 mL/d	1200mgx2 NAC po for 4 weeks	GFR, 24 h urine volume, Kt/V	Improvement of kidney function
Shahbazian et al. [2]	2019	Double-blind RCT	40 HD patients	600 mg × 2 NAC po for 6 weeks	TAC	Improvement of OS
Tsai et al. [108]	2010	RCT	43 high-flux HD patients with or without RRF	Addition of 5 g NAC IV to normal saline during HD session	Serum TNF-α, IL-10, hs-CRP, total Hcy	Decrease in total Hcy
Thaha et al. [106]	2006	Placebo-controlled RCT	60 HD patients	4 h NAC IV during HD session	Plasma Hcy, heart rate, pulse pressure	Decreased Hcy, improvement in pulse pressure
Scholze et al. [68]	2004	Placebo-controlled, cross-over RCT	20 HD patients	4 h NAC IV during HD session	Plasma Hcy, pulse waves during HD	Decreased Hcy, improvement in pulse pressure and endothelial function
Friedman [109]	2003	Placebo-controlled RCT	38 HD patients	1200mgx2 NAC po for 4 weeks	Hcy plasma levels	No effect
Perna et al. [66]	2012	Open, parallel	145 HD patients	MTHF +—5 g NAC IV during HD for 10 sessions	Hcy plasma levels	Decrease in Hcy
Bashardoust et al. [49]	2017	Placebo-controlled RCT	51 HD patients	1200 mg NAC po for 4 weeks	Hb, ferritin, hs-CRP	Improvement in anemia and inflammation
Bostom et al. [107]	1996	Prospective	11 HD patients	1 dose of 1200 mg po NAC	Hcy plasma levels	No effect
Modarresi et al. [9]	2017	Double-blind, placebo-controlled RCT	57 kidney transplant recipients	NAC po: 600 mg before- followed by twice daily up to the fifth day after transplantation	GPX activity, serum MDA levels, first week eGFR, graft function	No effect on GPX/MDA 28% better graft function, 14 ml/min higher eGFR
*PD*						
Nascimento et al. [117]	2010	Placebo-controlled clinical	30 PD patients	600 mg × 2 NAC po for 8 weeks	hs-CRP, IL-6, TNF-a, AOPPs, GSH, Hcy, ADMA, free sulfhydryls	Improvement of inflammation No effect on OS
Purwanto et al. [6]	2012	Placebo-controlled clinical	32 PD patients	600 mg × 2 NAC po for 8 weeks	PCT, IL-6, IL-1, C3, SICAM, hs-CRP, TNF-a	Improvement of inflammation
Feldman et al. [119]	2011	Prospective open-label, self-controlled	10 PD patients	1200 mg × 2 NAC po for 4 weeks	RRF, Urine volume Residual Renal Kt/V	Improvement
Najafi et al. [118]	2021	Quasi-experimental self-controlled	50 PD patients	600 mg × 2 NAC po for 8 weeks	hs-CRP	Improvement

A summary of clinical trials investigating the use of NAC in CKD, HD and PD assessing its antioxidant and anti-inflammatory properties

*ADMA* asymmetric dimethylarginine, *AOPPs* advanced oxidative protein products, *C3* complement C3, *CD11b/CD18* cluster of differentiation 11b/cluster of differentiation 18, *CKD* chronic kidney disease, *CV* cardiovascular, *GPX* glutathione peroxidase, *GSH* reduced glutathione, *GSSG* oxidized glutathione, *Hb* hemoglobin, *Hcy* homocysteine, *HD* hemodialysis, *hs-CRP* high-sensitivity C reacting protein, *IL-1* interleukin 1, *IL-10* interleukin 10, *IL-6* interleukin 6, *IL-8* interleukin 8, *IV* intravenous, *MDA* malondialdehyde, *MPO* myeloperoxidase, *MTHF* 5-methyltetrahydrofolate, *NO* nitrogen oxide, *OS* oxidative stress, *PCT* procalcitonin, *po* per os, *RAAS* renin–angiotensin–aldosterone system, *RCT* randomized controlled trial, *RRF* residual renal function, *sICAM-1* soluble intercellular adhesion molecule-1, *SOD* erythrocyte superoxide dismutase, *TAC* total antioxidant capacity, *TNF-α* tumor necrosis factor-a, *vWF* Von Willebrand factor

The antioxidant and anti-inflammatory effects of NAC in PD are currently supported mainly by experimental studies and, therefore, no recommendations regarding NAC administration can be supported in PD patients.

## Conclusions

In CKD, OS has emerged as a novel disease-related risk factor for CV disease, mortality, and progression to ESRD. In HD and PD, OS is further exacerbated and strongly associated with adverse clinical endpoints. NAC, a thiol compound, mostly known for its potential to reduce incidence of contrast-induced nephropathy has generated a lot of interest as an antioxidant agent and a potential candidate to combat OS-induced damage. Accumulated data suggest that in CKD, HD and PD patients, NAC neutralizes pro-oxidant molecules, increases antioxidant defenses, decreases Hcy, and suppresses inflammation, and thus might be beneficial for these patients. Moreover, limited data suggest that NAC might have beneficial impact on clinical hard endpoints in these populations, including protection of kidney function and prevention of endothelial dysfunction and CV disease. Of note, NAC is a safe agent without severe side effects, simple and of low cost. However, the data regarding the association of NAC with clinical hard points remain limited and derived from small studies with heterogenous populations. Well-designed RCTs with large sample size and hard endpoints are needed to draw definite conclusions regarding the beneficial effects of NAC in uremic populations.

## Data Availability

All data used for this article are available.
